# Politicizing Social Inequality: Competing Narratives From the Alternative for Germany and Left-Wing Movement *Stand Up*

**DOI:** 10.3389/fsoc.2020.00013

**Published:** 2020-03-11

**Authors:** Oliver Schmidtke

**Affiliations:** Department of Political Science, University of Victoria, Victoria, BC, Canada

**Keywords:** populism, social movement activists, democracy, Germany, social inequality, austerity

## Abstract

This article investigates the link between rising levels of social inequality and years of austerity on the one hand and the rise of populist, anti-establishment protest on the other. This connection is explored by analyzing the discursive practices of activists as a way of reconstructing the key argumentative and emotional structures organizing actors' understanding of politics. Empirically the article is based on 40 narrative interviews with supporters of the German right-wing, anti-immigrant party, *Alternative for Germany* (AfD), and the newly established left-wing movement *Stand Up*. The findings of the discursive analysis point to a profound sense of exclusion amongst left- and right-wing populist affiliates defined both in socio-economic terms and with a view to being deprived of a proper political voice. At the same time, the results show that the supporters of the AfD, in contrast to those from *Stand Up*, develop a strong, mobilizing collective identity that is instrumental in popularizing their discontent with the political establishment: The dramatized conflict between the virtuous German people and the threatening Other—manifested primarily by immigrants and the European Union—provides an emotionally charged binary that is at the core of the contemporary populist resurgence across Western democracies. In addition, the collective identity is instrumental in offering a particular interpretation of the origins of and desirable response to growing inequality that rely more on culturalist rather than traditional class-based arguments. Building on this analysis, the article offers an interpretation of the relative weakness of the populist left that, in the German context, so far has not succeeded in using deepening socio-economic cleavages for their political mobilization effectively.

## Introduction: the Challenge of Populist Resurgence

Social inequality is a fundamental driving force in defining political cleavages and conflicts. Since the Second World War, it has been the political left that has carried the struggle for greater social justice and equality. Yet, over the past decade and more vehemently during the last couple of years, the authoritarian, populist right has come to articulate and strategically mobilize the anger over a fundamental shift in Western societies that, as Piketty (2013) has demonstrated conclusively, has seen a substantial concentration of wealth and rising levels of inequality. As Barak Obama described in his speech from 2013, it is this disparity that is the “defining challenge of our times.”

The 2008/09 financial and economic crisis in particular has had profound effects on the social fabric of European societies as well as profound realignments of party politics in Western democracies (Kriesi, 2014; della Porta, 2015; Kriesi and Pappas, 2015; Giugni and Grasso, 2016). Manifestly, the impact of the *Great Recession* varies across the European continent: while the so-called PIIGS countries (Portugal, Ireland, Italy, Greece and Spain) have endured years of EU-imposed austerity regimes, declining living standards, high unemployment rates, and a gradual decline of the middle class (Guillén and Pavolini, 2015). Other EU member states, most notably Germany, have escaped the most severe economic and social repercussions of the crisis. Still, even in those more privileged countries the crisis has transformed European societies by accentuating class cleavages and by provoking significant discontent with mainstream politics (Hernández and Kriesi, 2016; Stavrakakis et al., 2018).

While the direct causal link is debated, there seems to be a clear correlation between the experience of prolonged austerity and growing inequality on the one hand, and the rise of anti-immigrant, nationalist-populist political forces across Europe on the other (Albertazzi and Mueller, 2017). Undermining the social contract that, arguably, had been in place for the post-war decades and accounted for political stability, has translated into a notable decline in popular trust toward the entire political class. Exploiting and fueling this alienation from the established elites, the specter of populism has developed into a veritable challenge for Western Europe's established liberal democracies (Hartleb, 2015; Greven, 2016). The success of populist, anti-establishment parties has further changed the dynamic of competitive party politics in a range of European countries radically. In recent elections across the continent, established centrist parties have been decimated and, in particular, the center left has lost much of its former electoral base (Hobolt and Tilley, 2016; Ramiro, 2016). In countries, such as France, Greece, or Italy, traditional electoral politics has been turned up-side down driven by a prise of new political actors and a rapid popular decline in the trust of mainstream politics and institutions.

The article tackles the broader research question why the traditional left has largely failed to capitalize on the economic crisis and why its political narrative has not spoken to the social sensitivities as effectively as the nationalist-populist one. Why have right-populists whose political program is regularly relatively unspecific about social and economic policies seen their political fortunes rise in the post-recession decade while their leftist counterparts whose political identity is fundamentally shaped by social justice concerns has been decisively less successful? Whereas in 2008, Decker still diagnosed “right wing populist failures and left wing successes” (see also Decker and Hartleb, 2007; Decker, 2008), this trend appears reversed in contemporary German politics.

To start, I will contextualize the German context 2-fold: First, I briefly address the conceptual discussion of populism as a response to a political situation in which considerable parts of the population experiences an alienation from established political institutions and modes of decision-making. In this perspective, I interpret populism from the left and the right as a viable option to address issues of social justice and equality based on a radical critique of liberal democracy (Canovan, 1999, 2002). Second, I discuss the rift between different groups on the left in Germany, most importantly the established parties and the newly formed social movement initiative “*Stand Up*” (in German *Aufstehen*) initiated to revitalize the progressive forces outside of the established institutional setting. While we do observe similar developments across Western democracies, these structural changes in electoral and party politics find their specific manifestations in national contexts.

To address this broader research question of the fractured left, I delve into an empirical study based on narrative interviews with supporters of the leftist *Stand Up* movement and of the *Alternative for Germany* (AfD), a right-wing party that, for the first time in Germany's post-war history, entered German Parliament in 2017 (Arzheimer, 2015; Bebnowski, 2015; Decker, 2016; Lees, 2018)^1^. Both political formations are situated in the political space between a social movement and a political party. Furthermore, they both show distinct forms of populist protest and anti-establishment mobilization. The main objective of analyzing the two sets of interviews is to establish in what way issues of austerity, marginalization, and social inequality steer perceptions and political orientations. This investigation will provide the grounds for a concluding reflection on the role of left- and right-wing populism in German politics and, from a sociological perspective, the way in which we can differentiate them by their respective discursive strategies to address austerity, the entrenchment of the welfare state, and growing social inequality (Flora and Heidenheimer, 2017).

In this latter respect, this article is also a contribution to the debate on the driving forces behind the populist resurgence in Western democracies: Can we indeed speak of a direct causal link between how economic risks and disadvantages translate into preferences for populist actors? With its focus on the discursive, ideational practice of affiliates of the populist left and right, I suggest a sociological reinterpretation of simplistic approaches in the political economy tradition (see Manow, 2018; Rodrik, 2018). Adopting this interpretive lens I also intend to add nuance to the overgeneralized claim that in particular those endorsing the populist, nativist right can solely be categorized as the “losers of globalization” (see Inglehart and Norris, 2016; Rooduijn, 2018; Koppetsch, 2019)^2^.

## Left vs. Right-Wing Populism: Contextualizing the German Case

The rise of populism in Western democracies is at its core a reflection of the challenges that democracy as a system of governance has faced over the past two decades. The late Peter Mair has been an authoritative voice in pointing to the profound crisis of democracy, a gradual hollowing out of the sovereigntist promise to its citizens: “… and the citizenry are becoming effectively *non-sovereign*. What we see emerging is a notion of democracy that is being steadily stripped of its popular component—democracy without a demos” (Mair, 2006: p. 25; see also Mair, 2013). In the scholarly community, there is growing recognition that liberal democracy suffers from an erosion of trust in the institutions and actors that have the mandate to represent the will of the people (see also Crouch, 2004; Plescia et al., 2019). In this respect, the populist surge is also a reflection of the declining trust and confidence that citizens have in traditional forms of representative democracy (Alonso et al., 2011), or as Berman (2019) calls it, a “symptom of growing dissatisfaction with democracy.”

At the core of the current populist surge is the claim to represent the *vox popoli*, the “voice of the people” defined by the dramatized contrast to the political elite or establishment (Barr, 2009; Stavrakakis, 2014)^3^. Hartleb has described this reference to the clash between the rulers and the ruled as a veritable cleavage transcending the traditional left-right divide and feeding populism's attractiveness. The ideological ambiguity (Albertazzi and McDonnell, 2008; Stanley, 2008) and popular appeal of populism make it an intellectually fascinating, albeit theoretically challenging subject of study. The conceptual uncertainty is rooted in the versatility of the claim to represent the interest of ordinary people in a direct and genuine manner. Mudde (2017: p. 33) and Stanley (2008) call populism a “thin-centered ideology” that is qualitatively different from other core political ideas (similarly: Manin, 1997; Muller, 2011). Populism is a mode of engaging in politics that is not exclusive to a particular ideological position or type of political actor (Moffitt, 2016). Rather, the form of political engagement—its reliance on direct political action, a strong mobilizing collective identity, and charismatic leadership—is the constitutive mark of populism (Albertazzi and McDonnell, 2008; Mudde and Kaltwasser, 2012; Moffitt and Tormey, 2014).

While extreme forms of nationalism, authoritarian leanings, and aversion toward the ethno-cultural other are an important element in the ideological reservoir of populist actors, such a perspective ignores the existence of leftist populism, which employs its own dichotomy between the “pure people” and the “corrupt elite” without having to rely on the hatred of foreigners or migrants (Rama and Santana, 2019)^4^. Under different theoretical auspices, Laclau and Mouffe see populism to be the very essence of grass-root democratic or “radical” politics with a veritable emancipatory claim (Laclau and Mouffe, 1985; Laclau, 2005; Mouffe, 2018, 2019). In Mouffe's interpretation, populism from the left is the signal of and appropriate response to the crisis of neoliberal hegemony and the growing social inequality it has promoted.

In this respect, populism functions as a mirror of the state of, or as Mudde and Kaltwasser (2012) phrase it, a “corrective of democracy”: Populism reflects the degree to which democratic institutions and processes can make a legitimate claim to provide the people with a credible and widely accepted political voice. This concern regarding the effectiveness or legitimacy of democracy is reflected in political subjectivities, citizens' way of interpreting socio-political realities and developing action strategies accordingly. In the 1980s, Dubiel (1986: p. 90) described the “populist moment” (Goodwyn, 1978) as one when larger social groups have, “collective experiences of felt offense,” and responding to a deep sense of social instability, embark on developing new political subjectivities. The subsequent empirical analysis of competing contemporary populist movements in Germany will focus on the way in which the populist promise of empowering the people and providing them with an authentic “voice” manifests itself in the perceptions and orientations of their respective supporters.

### Populist Politics in Germany: the Legacy of a De-radicalized West German Left

The legacy of the Cold War and the division of the country fundamentally shapes the recent history of the left in Germany. Shortly after the Second World War and the establishment of both German states, the Social Democratic Party (SPD) moved away from its class-based identity of the Weimar Republic and to the political center in the Federal Republic of Germany (FRG). With its 1959 Godesberger Program, the SPD reinvented itself as a so-called “people's party” that dropped its exclusive working-class orientation and embarked on a strategy of reforming the capitalist system rather than replacing it. Much earlier than in other European countries (most notably France, Greece, and Italy), the radical Socialist or Communist alternative disappeared in competitive party politics^5^. Mirroring the development of the Social Democratic Party, the working class and unions in the FRG largely de-radicalized and abandoned their revolutionary tradition (Berger, 2014).

When it comes to articulating anti-austerity sentiments there are three additional factors that has prevented the social democratic center left to become the political voice for those who have suffered from or are opposed to cuts to the welfare system or neoliberal deregulation of the economy: First, the German SPD became part of the New Labor camp starting in the 1990s (prominently led by the UK Labor under Tony Blair; see Schmidtke, 2002). The principal political orientation of this iteration of social democratic politics was an increasing emphasis on the individual and a general mistrust in the regulative capacity of the state. Accordingly, under Chancellor Gerhard Schroeder, the SPD launched the so-called agenda 2010 that, at its core, promoted market liberalization in the early 2000s. In the name of enhancing Germany's competitiveness in the global market, the SPD-led government introduced tax cuts, deregulations to the labor market, and major cutbacks to the social systems (pension benefits, medical services, etc.)^6^. Since this time, the SPD has struggled to present itself as an advocate of the “ordinary people” and political champion of social justice.

Second, regarding the issue of austerity and social inequality there are two dominant lines of conflict: One cleavage is structured primarily along class lines. As in other countries in the Western world, Germany has witnessed a deepening social inequality over the past two decades^7^. Phenomena, such as the working poor are relatively new and have come to shape the country noticeably. In addition, there is the regional divide in particular between the relative prosperous Western part and the relative deprived Eastern part of the country^8^. It is against this background that the “Linke” has established itself both as a leftist, socialist organization with a strong class base and as an advocate for the citizens of the former GDR many of whom feel marginalized and not properly recognized in united Germany. In the German context, social inequality is also forcefully coded as a regionalist conflict and one fundamentally characterized by the legacy of the pre-1989 period.

Third, since 2013 the SPD has been part of the so-called Grand Coalition under the leadership of Chancellor Merkel. In this role, the German center-left party has struggled to live up to its own claim to be the “party of social justice.” While the SPD could introduce legislation to address issues of austerity and social inequality (such as a raising minimum hourly wages, providing more affordable child care, implementing initiatives for a basic income, etc.), its profile in this respect has been overshadowed by the image of a government that has lost its willingness or ability to take on challenging decisions and develop daring policy initiatives (Schmidtke, 2016). Having the Grand Coalition in power for such a long time has provided the ground for the popular perception of a power monopoly of the main parties and the lack of an effective opposition (Bremer, 2017).

It is against this background that the SPD has become fundamentally challenged in its claim to represent the progressive option in German politics. In the last federal election, the social democrats received the lowest support since the foundation of the FRG in 1949: the 20.5% is far away from the high 30% or low 40% range that the SPD could count on during the decades leading up to the early 2000s. The latest surveys indicate a further loss in support, putting the SPD as a “people's party” behind the Greens (and the AfD) as a minor opposition party. This dramatic loss in support and trust has sparked the formation of a new political formation that seeks to give a new voice to the German left: “*Aufstehen*” or “*Stand Up*.”

### Challenging the Established Leftist Agenda—Toward a New Leftist Social Movement?

The eroding trust in the *Social Democratic Party* and the rise of the *Alternative for Germany* has set the agenda for a left-wing social movement. Officially launched in September 2018, *Stand Up* was initiated by a group of 80 authors, artists and professors with the goal to “bundle” left-wing efforts and set the agenda for a future leftist federal government. As one of the co-initiators of this new political actor Sarah Wagenknecht, a prominent politician from the “Left” Party, declared that the main political objective of *Aufstehen* is to regulate “neoliberal capitalism.” The growing social inequality is key and center of its campaign: “People working under short-term contracts, or with too small pensions, and with children that can no longer receive a decent education because the public schools are falling apart and don't have enough teachers, they have every reason to be angry at ‘those above”'^9^.

Another reason for forming the movement—next to the widely shared dissatisfaction with the left—was the perceived need to address the rise of the extreme right in Germany. According to Wagenknecht, it is a “deep crisis of democracy” that is the driving force behind the popularity of the protest vote for the extreme right, most prominently PEGIDA^10^ and the Alternative for Germany. *Stand Up* claims to vocalize the grievances of disaffected citizens who suffer from an economy that is portrayed as not serving the people (Vorländer et al., 2018). In her speeches, Wagenknecht makes the direct link between years of austerity measures, growing social inequality and the crisis of democracy: “Despite economic growth, 40 percent of residents have less net income than 20 years ago; democracy is no longer working”^11^.

The decision to form a broader movement outside of Parliament and the party system (at the time Sarah Wagenknecht was the co-leader of the “Left” Party), was born out of frustration with the current German left, and the perceived mismatch between the popularity of “leftist” ideas and the relative marginalization of leftist parties in contemporary electoral politics. *Stand Up* was conceived of as a broad social alliance on the left and was meant to replicate the success of the radical, grassroots left like the French Left Party under Jean Luc Mélenchon or Podemos led by Pablo Iglesias in Spain (Kioupkiolis, 2016; Ivaldi et al., 2017; Ramiro and Gomez, 2017). By mid 2019, the alliance claimed that *Stand Up* had signed up 101,000 adherents.

Yet it is worth pointing out that, at this moment (the fall of 2019), the initial excitement within leftist circles has waned considerably. Thus far, *Stand Up* has not developed the momentum of, for instance, the Yellow Vests in France. The growing reservation comes primarily from the established parties on the left (the Left and the SPD) that are uncertain about what relationship such a grassroots-oriented and participatory movement is supposed to have with the traditional agents of representative democracy. In addition, Sarah Wagenknecht was heavily criticized when she refused to take part in the demonstration “*Unteilbar*”^12^ (Indivisible) which, with over 300,000 participants, was organized in support of an open, inclusive society and directed against the anti-immigrant rhetoric of the right in October 2018. Wagenknecht accused the organizers of promoting “open borders” that, in her view, would be incompatible with protecting the German labor market from unregulated immigration. As I will point out more fully in the next section, issues of borders and migration should play a central role in the political worldviews of the activist from the AfD and *Stand Up*.

## Materials and Methods: Narrative Interviews With Supporters From Two Competing Populist Political Groups

This study is based on two sets of interviews conducted with supporters^13^ of the right-wing *Alternative for Germany* and the leftist movement *Stand Up* (interviews were organized in Berlin and Dresden in the fall of 2018 and the spring of 2019). These two political actors hold opposing ideological views but are driven by a comparable claim to give voice to those who have been marginalized or excluded in politics. Methodologically the investigation was guided by the design of narrative interviews in the qualitative research tradition that seeks to encourage longer, narrative accounts from the interviewees based on open ended questions (in this case, their perspectives on contemporary political challenges, institutions and actors). In addition, the respondents of both groups were asked to fill out a simple survey in which they were invited to indicate their trust in major political and social institutions using one of four categories (trust fully, trust somewhat, mistrust somewhat, mistrust fully) as well as a self-categorization in terms of political ideology (left, right, or none). These categorizations were also used to open the narrative interviews and provide interviewees an opportunity to engage in a reflection that was steered by their own priorities and convictions.

This study follows the qualitative tradition of small case studies, which emphasizes conceptualizing the complex political-cultural context and being open to generating new theoretical insight driven by empirical findings (Maxwell, 2004; George and Bennett, 2005; Creswell, 2013). Narrative interviews provide us with an effective way of reconstructing the key argumentative and emotional structures organizing actors' understanding of politics. The set of 40 interviews was coded based on central themes and frames as underlying organizing principles of the narration. The qualitative interpretation of narrative interviews (see: Clandinin, 2006; Nohl, 2010) not only aims to reconstruct the explicit worldviews and political convictions of the interviewees. The method also enables us to understand the implicit set of knowledge and routines that shape and are embedded in discursive practices^14^. In this article, the analysis of the interviewees' narrations is meant to shed light on how the issue of austerity and growing social inequality is perceived, evaluated and included into a broader framework of political convictions and action strategies.

The research team coded the two set of interviews according to dominant themes that structure and organize the narration of the respondent. This approach to textual analysis allows for interpreting meaning through identifying central thematic structures and socio-political referents. The principal research question is directed at the link between austerity and social inequality on the one hand and the political preferences and worldviews of the interviewees associated with the respective populist actor on the other. Does the experience or fear of socio-economic decline and marginalization drive loyalty to the radical left and radical right in Germany? How and in what narrative context is the issue of socio-economic inequality and austerity framed politically? Based on this particular interpretative inquiry into the political subjectivities of populist parties' supporters, the central hypothesis addressed in the empirical analysis is the following: While supporters from the populist left and right share a profound sense of deprivation, the experience and perception of social inequality is couched in broader narratives of collective identity and cultural belonging.

**Figure 1:**
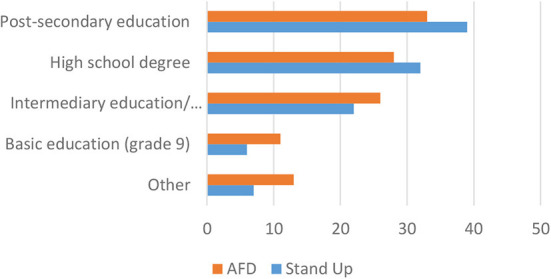
Educational background (in %).

We conducted the sampling of supporters with the aim to have comparable groups in terms of basic social characteristics. As is documented in Figure 1, the two groups are comparable with respect to educational background. Overall, the supporters of the leftist *Stand Up* movement are more highly educated in particular with respect to completing a university undergraduate program. Yet, both groups do not deviate majorly from the average educational achievements of Germans in general.

**Figure 2:**
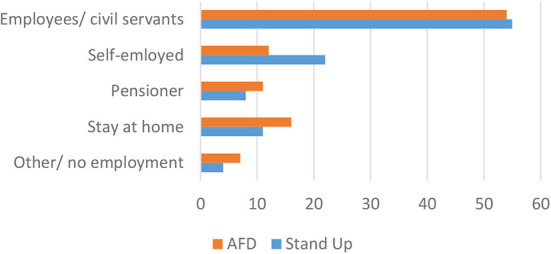
Professional status (in %).

Interestingly, there is greater variation when it comes to the professional status of the interviewees. As is documented in Figure 2, there is a higher representation of pensioners and stay-at-home women/men for the AfD and of students and self-employed for the *Stand Up* movement. Still, the sample is similar enough to assess the findings of the interviews in a comparative fashion without neglecting the role that the professional life of the respondents play in shaping their worldviews. Similarly, the two sample groups are comparable in terms of other social variables, such as gender (both groups have 10 male and 10 female interviewees) and age (within 3.4 years, the average age is slightly higher for the respondents from the ADF).

## Results: Competing Narratives—Inequality and Austerity in the Narratives of the Left and Right-Wing Populists

The initial survey of the 40 interviewees shed light on a differentiation between the two groups that will be key to interpreting the more detailed findings of coding the interviews: When the interviewees were asked whether they self-identify as left or right, the group of the *Stand Up* supporters was far more definitive in situating their *Weltanschauung* on the left-right axis. While those affiliated with *Stand Up* explicitly identified to be on the left (83%) and regularly used the reference to (democratic) socialism, the AfD supporters were far more hesitant to locate themselves on the right (54%). For them, the traditional ideological binary does not seem to be the exclusive or most adequate way to describe their political identity. Nationalism and the AfD's approach to identity politics sits uncomfortably with a political framework defined by issues of social inequality defined by traditional class relationships.

### Profound Mistrust in Traditional Political Institutions and Actors

**Figure 3:**
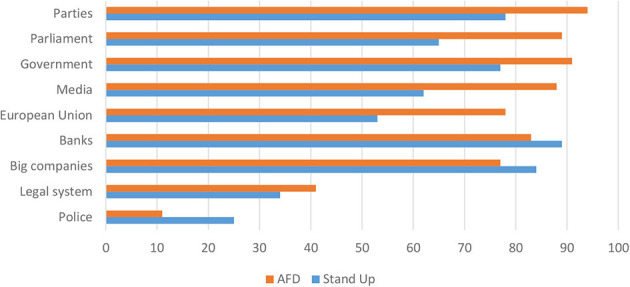
Mistrust in major institutions (in %).

One of the most pertinent themes and sensitivities that had a determining effect on the framing of members from both groups is the profound sense of not having a proper voice in politics and being “betrayed” by those in power. The introductory short survey with interviewees provides a first indication of this alienation from established institutions and actors in politics. As evident in Figure 3, the supporters of the AfD in particular show a persistent mistrust in any institution in representative democracy, be it domestic in terms of mainstream parties, parliament or the government or be it concerning the European Union (around 90% declared to mistrust somewhat or fully national institutions, close to 80% for the EU). For the supporters of the leftist movement, these numbers are also high (in the 55–75% range) indicating an articulate sense of not being able to trust nor feel represented by the democratic political system or its representatives.

Yet, it is noteworthy that interviewees from the left demonstrate a lesser degree of mistrust in the European Union. Given that both groups were chosen with a view to representing a populist opposition, these numbers might not be too surprising; yet the extent to which the trust in these institutions has eroded is worth underlining especially with a view to the central claim of populist actors to be deprived of a “voice” in public debate and political decision-making. In the interviews, it also became apparent that the supporters of both organizations insisted on representing the “will of the people” in a way that is fundamentally different from established parties and officials. It is at the very core of *Stand Up* to galvanize grassroots mobilization as an alternative mode of political engagement than the one offered by party politics.

The findings of the survey also point to how in particular the supporters of the AfD have a very limited trust in the media. Almost 90% of this group—compared to just over 60% from the *Stand Up* followers—do not trust different mass media (most significantly television and the quality print press). With respect to the main research question of this article directed at the role of austerity and social inequality as a driving force in propelling populist politics, the intense mistrust toward major representatives from the corporate world is worth highlighting. Here the members of the leftist group show an overwhelming mistrust in big companies and banks. However, the supporters of the AfD are relatively similar in their attitude toward the big players in business. The legal system and in particular the police command a substantially higher degree of trust than the other public or private institutions.

**Figure 4:**
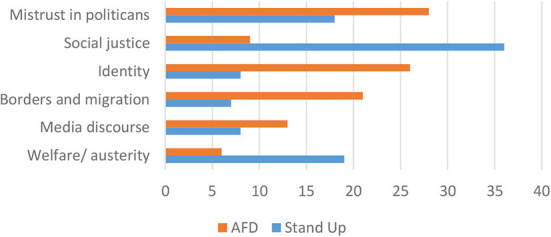
Thematic fields addressed in narrative interviews (in %).

The narrative interviews underlined the central issue of mistrust toward or at times open contempt for the fundamental institutions of representative democracy. Repeated reference was made to “those up there” who do “whatever they please” and are “unable or unwilling to listen.” As one interviewee from the AfD supporters put it dramatically: “They have stopped listening long ago. It is time to let those in power to find out what the people really want” (Interview AfD 4). This binary between the widely held convictions of the “ordinary people” and the political establishment (most notably the government under Chancellor Merkel) is thematic throughout almost all the interviews. There is a deep-seated sense of deprivation in terms of being recognized or listened to by those who make the decisions in politics and society. Again, an AfD supporter put it in the following way: “They simply don't care. They should serve us as the people—but they just look at us with contempt” (Interview AfD 12)^15^. As is also confirmed by the sheer emphasis put on this theme (see Figure 4 below), the mistrust in politicians, the notion of being “betrayed” by the establishment is constitutive for the worldview in particular of AfD loyalists.

The mistrust toward media is also far more pronounced among the supporters of the AfD than for those from the *Stand Up* movement. Media are characterized as being an integral part of the power elite that, with its alleged leftist-liberal bias, is accused of simply being a mouthpiece of the ruling elites. It is through this interpretative lens that media discourse also constitutes one of the central thematic fields in the interviews especially of the AfD (see Figure 4). Almost all media are depicted as sheltering public discourse from the legitimate concerns of ordinary citizens that are portrayed not to have a proper voice.

This point of utter estrangement with those in power (in the narration of AfD supporters, government and leftist-liberal media are often interchangeable in the discourse of the interviewees from the right) is prominently articulated with reference to issues of migration and more specifically the German response to the refugee crisis. In this respect, an anti-elitist agenda and deeply held anti-immigrant sentiments are mutually reinforcing and emotionally charged features of the narrative detected in the interviews. As one of the AfD affiliates stated: “Nobody wanted these refugees. Merkel simply allowed them in. You cannot enforce this on us ….” (Interview AfD 18). Settling asylum seekers in the wake of Germany's exceptional intake of refugees the 2015/16 is also a recurrent reference point in underlining the gulf between ordinary people and the elite: “The politicians do not have to live with all these refugees. My parents' village had to accept dozens and dozens of refugees. And there are no jobs to begin with …. This all does not make any sense.” (Interview AfD 6). The claim is that elites do not only fail to listen to the people, they are also portrayed to take decisions whose social repercussions have to be shouldered exclusively by regular people.

### Framing Populist Concerns Thematically

Figure 4^16^ summarizes the degree to which particular thematic fields were addressed in the interviews with the two groups. One of the key issues for the AfD supporters are migration and borders. These themes resonate strongly with the underlying nativist ideology of the party and, more important for the central research question of this article, the politically prominent plea to restore the “rights of the sovereign people.” The vividly described violation of border security is a regular narrative trope in the interviews with this group. It illustrates the organizing reference point for their worldview that the fundamental interests of the people are demarcated by national borders (security, economic well-being, etc.). It is noteworthy that socio-economic threats and the risks associated with migrants are interpreted based on the same central argument: The globalizing economy and migration pose challenges to the well-being of citizens that the state needs to respond to by protecting the integrity of these borders. The idea of “losing control,” of no longer guaranteeing border security is a central and emotionally charged image in the narrative repertoire of AfD supporters. In their narrative, borders are a critical political reference point or symbolic marker representing the fundamental challenges associated with a globalizing world and the promise for security and prosperity.

For those interviewees affiliated with the leftist movement *Stand Up*, the issue of border and migration takes up far less pronounced role. Reference to migrants is negligible while the issue of borders is addressed primarily as one associated with the dominance of global economic and financial power structures. The dominant theme in this respect is how national communities can protect the rights and social entitlements when they are under threat by the leveling forces of “global capitalism”: “We cannot go on like this. It is a constant race to the bottom. Well-paying jobs have replaced low paying ones due to companies exploiting the global economy” (Interview *Stand Up* 5). In the narrative of this group, the financial and economic crisis of 2008/09 is a constant reference point as well as a mobilizing source of frustration over the lack of political will to regulate financial and corporate power: “We play to the tunes of these big corporations. How have they gotten away with amassing their profits and get our bailouts when things got tough …. People need to take back control” (Interview *Stand Up* 7).

In the interviews with the *Stand Up* group, the specifics of austerity politics and the retrenchment of the welfare state came up repeatedly. The phenomenon of the working poor, the inadequacy of the German social security system, unaffordable housing as well as the uneven economic development of German regions featured prominently in the interviewees' narrative. Yet this group was even more outspoken about the larger issue of the growing social inequality in Germany: “The wealthy have become absurdly rich and the underprivileged have become poorer. This is unsustainable I don't want to live in a society like this” (Interview *Stand Up* 3). Several interviewees interpreted the 2008/9 crisis and its socio-economic effects in terms of a longer trend accentuating social divisions and inequalities. In this context, the established center left is accused of complicity in making neoliberal ideology dominant in society. The interviewees overwhelmingly attribute the rising levels of social inequality to the logic of capitalism and adopt a class centered perspective in depicting the growing gulf between rich and poor.

Supporters of the AfD express concerns regarding social deprivation along class lines in a more multi-dimensional manner. Not dissimilar from their leftist counterparts, there is a prevailing sense of being treated unfairly by the forces of globalization. Throughout the interviews with this group, there was a strong sense that the economic certainty and stability of the past has been lost. It is the fear of an uncertain future and the perception of no longer having a proper place in the fast changing globalizing economy, rather than the immediate personal experience of socio-economic deprivation, that significantly shapes the perception of the interviewed AfD affiliates: “We do not know what the future brings. One thing is certain: Our children will no longer have the kinds of jobs we are used to” (Interview AfD 9).

In spite of this profound sense of uncertainty, there is no “class consciousness” defined primarily as shared experiences in the work force. As other studies have underlined (see Goerres et al., 2018), the supporters of the AfD are highly diverse in their socio-economic status; they range from the precarious worker in the service sector to well-paid engineers in the automotive industry or relatively prosperous retirees. The elements that link the fears of future social decline and the political project of the AfD is the shared sense of pride in one's work (“honest” and “hard” labor) and the loyalty of the national economy. The political promise to protect jobs from the uncertainties of the global market provides the basis for collective action. This way of addressing issues related to social justice can also provide an interpretation of the relatively small amount of outright emphasis that the AfD supporters put on issues of related to social justice and in particular welfare/austerity (see Figure 4). For instance, in the interviews with this group, there was—surprisingly—little evidence of a sense of deprivation directly linked to concrete cuts to welfare programs or austerity programs. Rather, affiliates of the AfD expressed a generalized feeling of uncertainty and unease that does not easily fit into conventional categories of social inequality or exclusion.

### The Prominence of Identity Politics

It is worth stressing that the claim about being “left out” and the power of the “rich” can also be found in the narrative accounts of the AfD affiliates. Yet two features are worth noting in how the experience or fear of social marginalization is framed in the discourse of AfD supporters: First, the issue of social injustice is regularly depicted in terms of a resentment of many people in East Germany toward the more prosperous and powerful western part of the country. The following statement by an interviewee in Dresden reflects a recurrent discursive element in this group's discursive practice: “Whatever they promised us (after unification, O.S.) …. Now, where have the jobs gone to? My children cannot find employment here. We look like the shadow of our former self” (Interview AfD 19).

Second, the binary between the corrupt and unresponsive elite and the virtuous people is not primarily framed as a cleavage rooted in socio-economic configurations but as a culturally coded conflict between groups and their identities. The ADF interviewees articulately direct their fear of social decline and loss of status chiefly toward those who are portrayed to betray the interest and identity of the German population. One of the members of the AfD group puts this as follows: “Since all these foreigners have come nothing is the same anymore. They take the jobs, they receive our tax euros. I no longer recognize my neighborhood” (AfD Interview 7). The reference to an idealized past when life was secure, protected and prosperous is one of the recurrent and powerful reference points in the narrations of the AfD interviewees. Their accounts reflect a threatening image of far-reaching change in their lifeworld over which people are described to have lost control: “I am not sure what happened in our city. When I walk down the street, everything seems to have changed. This is no longer my place …” (Interview AfD 20). It is worth stressing that in the interviews with AfD affiliates notions of socio-economic deprivation are closely intertwined with a deep sense of cultural alienation and non-recognition. As another AfD supporter puts it: “This used to be my place. Nobody cares anymore what we want” (Interview AfD 7).

One recurrent argument in the interviews with AfD supporters is the link between the experience or fear of social decline and the presence of foreigners in society. In the interviews, the resentment with respect to the loss of socio-economic status is simultaneously directed at the social, economic and intellectual elites and immigrants or minorities. It is in this context that the EU is primarily attacked: It represents the world of remote bureaucratic elites and, at the same time, the agency that promotes a borderless, pluri-cultural Europe. The common reference point is the insistence on protecting the rights and privileges of the people defined in ethno-national terms: the “Germans” or the “German nation”. Koppetsch (2019: p. 217) speaks in this context of the populist promise of being “collectively re-sovereignized.”

## Discussion: the Attraction of Right Wing Populism

This article provides one investigative avenue into the question how austerity under neoliberal auspices and growing social inequality have contributed to the rise of the populist actors. The analytical lens developed here is to shed light on the link between the formation of political subjectivities through discursive practices and mobilizing efforts of the populist forces from the left and the right. The anger about socio-political realities becomes relevant for individual preferences and action strategies if it finds vindication in collectively shared belief systems and modes of interpreting these realities.

The findings from the narrative interviews with supporters of the right-wing AfD and the left-wing *Stand Up* provide a confirmation and further qualification of the initial hypothesis of this article. The analysis of the political subjectivities articulated by these two groups point to how the experience of growing social inequality and the effects of the Great Recession are framed in particular broader narratives of collective identity and cultural belonging. With respect to politicizing these experiences, it is worth bearing in mind that there are some similar patterns in how supporters of both political groups describe the rationale and objectives of their respective populist protest: a staunch rejection of and even contempt for traditional elites, a profound mistrust in established political institutions and actors, and a multifaceted, albeit vague demand for more direct decision making power granted to the “people.” The experience of the retrenchment of the welfare state and growing social inequality have created an environment in which populist actors call into question the *modus operandi* of traditional representative democracy.

Yet there are critical differences between the narrative accounts provided by the left and right-wing populists. Most significantly, the affiliates of the AfD link their account of socio-economic grievances with a robust collective identity that is both, rooted in a sense of relative material deprivation and in a notion of cultural exclusion from the establishment. This feature of a unifying, emotionally charged identity speaks to the very rationale of populism. This collective identity provides a tangible sense of the virtuous “Self” (defined primarily in ethnic or cultural terms) and, at the same time, a clearly identifiable and threatening “Other,” mainly identified as the foreigners and, most dramatically, refugees from the Global South. In this regard, it is very much part of the solidifying political identity of the AfD that, as Arzheimer and Berning's (2019) study of the party's electorate underlines, the salience of immigration has increasingly shaped the AfD's electoral appeal. A strong sense of identity and community built on anti-immigrant sentiments is critical for the mobilizing capacity of the AfD. This collective identity is instrumental in turning the perceived social and cultural marginalization into a vehicle of political protest and provide the yearning for belonging with a notion of a romanticized past when this identity was supposed be pure and untainted. On various occasions interviewees from this group emphasize that this protest party provided them with a new “political home.”

More specifically, the collective identity based on a clear sense of “Us” (the locals, the Germans) and “Them” (the foreigners, the EU) is critical for the mobilizing efforts of the AfD in three ways. First, the strong collective identity promises itself to provide a remedy against the experience of social decline or marginalization: pride in the national community and the promise of solidarity based on a nativist identity. Salmela and von Scheve (2017) describe how, from a social-psychological perspective, right-wing populists offer a politically effectual strategy to address the fear of social decline and status inconsistency. Their underlying collective identity provides an ideational avenue to transform uncertainty and fear into resentment and hatred toward the perceived enemy of the people. Using the ethnic or cultural “Other” as a scapegoat for social ills is as emotionally exhilarating as politically shrewd. This reliance on a strong, predominantly ethno-centric Us-vs.-Them binary is at the core of the AfD's mobilizing strategies (see Greven, 2016). In this respect, Rensmann's (2018) diagnosis that the political radicalization of the party is not detrimental to its popular appeal points to how central discourses of othering and exclusionary nationalism are to the recent electoral successes of the AfD.

Second, given the nature of AfD and *Stand Up* as political formations claiming to be both a social movement and a party (or at least having aspirations for contributing to or even forming government), the issue of a mobilizing collective identity is critical. Parties compete in elections and try to win public office. In contrast, social movements focus on mobilizing public support and offering interpretative frames for particular, politically controversial issues. Given their grassroots mode of organization, social movements are dependent on a strong unifying and mobilizing collective identity (Goldstone, 2003; Rucht, 2019). The interviews with AfD supporters underline the imminent mobilizing force of its collective identity that speaks directly to the nature of the populist mode of politicization: Its nationalist and nativist rhetoric establishes the effective image of a coherent political agent united by fundamentally shared interests and cultural bonds. In her recent book, Koppetsch (2019) speaks about right-populism in the context of a “society of anger.” It is the ability of these populist actors to inflame this anger and steer it in a way to form political loyalties. A strong collective identity is essential to give this anger a political direction and to mobilize those who feel alienated from the political mainstream.

Third, a strong nativist ideology can function effectively as an interpretative lens for collective action frames of populist movements: Based on the emotionally charged “Us” vs. “Them” binary, right wing populists can promote simplistic political goals and policy objectives for complex social challenges (for instance, addressing unemployment by closing borders to foreigners). Populists from the right are effective in redefining interests based on socio-economic conditions by reference to primary group allegiance. The experience of marginalization and economic hardship in terms of class is reinterpreted and modified as the deprivation of an ethno-culturally defined group. The suggested homogeneity of “the Germans” or “the foreigners” creates clear-cut categories of interests and entitlements in policy making.

This strong collective identity from the populist right underpinning its political mobilization is reminiscent of the traditional class-based identity of the working class movement. It is worth underlining that in terms of framing years of austerity and deepening social inequality, a traditional class perspective and a related account of the power imbalances in contemporary capitalism constitute the narrative account of the interviewees from the left. Yet, in the discourse of the *Stand Up* supporters, this class perspective is expressed in rather general terms providing the basic rationale of the populist binary with adversaries at a relatively high level of abstraction (the “capitalist system,” “the 1%,” “big banks,” etc.). Similarly, the collective agent defined by a shared goal and collective identity remains rather vague in the narrative accounts of the interviewees. This finding can provide some clues in tackling the initial question about the weakness of the left in exploiting the growing inequality for the purpose of political mobilization. As this new political formation from Germany shows, the populist left finds it challenging to compete with the emotional energy of the mobilizing collective identity and the (over-) simplicity of the political messaging of the populist right.

## Data Availability Statement

The raw data supporting the conclusions of this article will be made available by the authors, without undue reservation, to any qualified researcher.

## Ethics Statement

The studies involving human participants were reviewed and approved by University of Victoria. The patients/participants provided their written informed consent to participate in this study.

## Author Contributions

The author confirms being the sole contributor of this work and has approved it for publication.

### Conflict of Interest

The author declares that the research was conducted in the absence of any commercial or financial relationships that could be construed as a potential conflict of interest.

## Footnotes

^1^I would like to acknowledge that the research on which this article is based has been supported by the Social Sciences and Humanities Research Council of Canada as well as by the Jean Monnet Program of the European Union.

^2^Indeed, there is sound empirical evidence from the German context to suggest that the supporters of the AfD are not necessarily those who are most economically deprived (Goerres et al., 2018).

^3^Albertazzi and McDonnell (2008: p. 3) define populism as “an ideology which pits a virtuous and homogeneous people against a set of elites and dangerous “others” who are together depicted as depriving (or attempting to deprive) the sovereign people of their rights, values, prosperity, identity and voice.”

^4^Recent empirical studies suggest that the left- and right wing positions toward and effects on democracy are distinct and essentially shaped by the opposed political ideology of both political forces (see Otjes and Louwerse, 2015; Huber and Schimpf, 2017; March, 2017).

^5^The Communist Party of Germany was banned as extremist in West Germany in 1956 and only reappeared under a new name as a fringe party in German politics after 1969.

^6^As a consequence, wages in Germany have shown one of the slowest growth rates in Europe and the number of low-paying, precarious jobs has grown exponentially over the past 15 years.

^7^According to World Bank data, ranking Germany 12th with a relatively low GINI coefficient (measuring levels of social inequality). However, since the 1990 the gap between the rich and poor has been growing. Recently the German Institute for Economic Research reported that Germany's wealth distribution is the most unequal one in the Euro zone: https://www.reuters.com/article/us-germany-wealth/germanys-wealth-distribution-most-unequal-in-euro-zone-study-idUSBREA1P1VJ20140226.

^8^The reality is more complex than the image of a west-east divide (there are successful economic centers in the east and parts of Western Germany that face major economic challenges). Still in public discourse, this cleavage is politically most powerful (see Olsen, 2018).

^9^Speech by Sarah Wagenknecht on August 11, 2018 (translated statement available at: https://www.dw.com/en/germany-new-aufstehen-movement-of-sahra-wagenknecht-is-shaking-up-leftists/a-45047762)

^10^PEGIDA is the *Patriotic Europeans Against the Islamisation of the West* movement that is built on strong anti-Islam, anti-migrant sentiments.

^11^Speech by Sarah Wagenknecht on September 4, 2018 (translated statement available at: https://www.dw.com/en/cross-party-leftist-alliance-launched-in-germany/a-45344972-0).

^12^See https://www.unteilbar.org/

^13^These supporters can also only have a loose connection to the organization of the two political actors. In the case of the AfD sympathizers, less than half were party members and for *Stand Up* only 30% of the interviewees signed up for the movement.

^14^See on studies following a similar analytical focus on discursive practice in studying populist parties and movements (Caiani and Kroll, 2017).

^15^With a view to this dominant narrative trope (not being taken seriously; being deprived of any relevant voice) takes on a particular meaning in the East of Germany where the AfD is far more successful electorally than in the Western states. In the former GDR there is a widespread sense of alienation from the West German institutions and of being treated in a patronizing way by those in power in the unified Germany (see Patton, 2017).

^16^The total of the percentages do not add up to 100%. The overview does not reflect other thematic issues that only received relatively little attention in the interviews.

## References

[B1] Albertazzi D. McDonnell D. (2008). Twenty-First Century Populism. London, UK: Palgrave Macmillan.

[B2] Albertazzi D. Mueller S. (2017). “Populism and liberal democracy: populists in government in Austria, Italy, Poland, and Switzerland,” in: The Populist Radical Right: A Reader, ed Mudde C. (New York, NY: Routledge, 508–526.

[B3] Alonso S. Keane J. Merkel W. (Eds.). (2011). The Future of Representative Democracy. Cambridge: Cambridge University Press.

[B4] Arzheimer K. (2015). The AfD: finally a successful right-wing populist eurosceptic party for germany? West Eur. Polit. 38, 535–56. 10.1080/01402382.2015.1004230

[B5] Arzheimer K. Berning C. C. (2019). How the alternative for Germany (AfD) and their voters veered to the radical right, 2013–2017. Elect. Stud. 60:102040. 10.1016/j.electstud.2019.04.004

[B6] Barr R. R. (2009). Populists, outsiders and anti-establishment politics. Party Politics. 15, 29–48. 10.1177/1354068808097890

[B7] Bebnowski D. (2015). Die Alternative für Deutschland: Aufstieg und gesellschaftliche Repräsentanz einer rechten populistischen Partei. Wiesbaden: Springer VS.

[B8] Berger S. (2014). Social Democracy and the Working Class: In Nineteenth-and Twentieth-Century Germany. New York, NY: Routledge.

[B9] Berman S. (2019). Populism is a symptom rather than a cause: democratic disconnect, the decline of the center-left, and the rise of populism in western europe. Polity 51, 654–667. 10.1086/705378

[B10] Bremer B. (2017). The Crisis of the SPD: Where Now for Germany's Social Democrats? London: LSE European Politics and Policy (EUROPP) Blog, 1–5.

[B11] Caiani M. Kroll P. (2017). Nationalism and populism in radical right discourses in italy and germany. Javnost Public 24, 1–19. 10.1080/13183222.2017.1330084

[B12] Canovan M. (1999). Trust the people! populism and the two faces of democracy. Polit. Stud. 47, 2–16. 10.1111/1467-9248.00184

[B13] Canovan M. (2002). “Taking politics to the people: populism as the ideology of democracy,” in: Democracies and Populist Challenge, eds Meny Y. Surel Y. (London: Palgrave MacMillan), 25–44. 10.1057/9781403920072_2

[B14] Clandinin D. J. (Eds.). (2006). Handbook of Narrative Inquiry: Mapping a Methodology. Thousand Oaks, CA: Sage.

[B15] Creswell J. W. (2013). Qualitative Inquiry and Research Design: Choosing Among Five Approaches. Los Angeles, CA: SAGE Publications.

[B16] Crouch C. (2004). Post-democracy. Cambridge: Polity.

[B17] Decker F. (2008). “Germany: right-wing populist failures and left-wing successes,” in Twenty-First Century Populism, eds Albertazzi D. McDonnell D. (London: Palgrave Macmillan), 119–134.

[B18] Decker F. (2016). The “Alternative for Germany:” factors behind its emergence and profile of a new right-wing populist party. German Polit. Soc. 34, 1–16. 10.3167/gps.2016.340201

[B19] Decker F. Hartleb F. (2007). Populism on difficult terrain: the right-and left-wing challenger parties in the Federal Republic of Germany. German Polit. 16, 434–454. 10.1080/09644000701652466

[B20] della Porta D. (2015). Anti-austerity Protests in the Crisis of Late Neoliberalism. Cambridge: Polity.

[B21] Dubiel H. (1986). The specter of populism. Berkeley J. Sociol. 31, 79–91.

[B22] Flora P. Heidenheimer A. J. (2017). “The historical core and changing boundaries of the welfare state,” in Development of Welfare States in Europe and America, ed Flora P. (New York, NY: Routledge), 17–34. 10.4324/9781351304924-3

[B23] George A. L. Bennett A. (2005). Case Studies and Theory Development. Cambridge: MIT Press.

[B24] Giugni M. Grasso M. T. (2016). Austerity and Protest: Popular Contention in Times of Economic Crisis. New York, NY: Routledge.

[B25] Goerres A. Spies D. C. Kumlin S. (2018). The electoral supporter base of the Alternative for Germany. Swiss Polit. Sci. Rev. 24, 246–269. 10.1111/spsr.12306

[B26] Goldstone J. A. (Eds.). (2003). States, Parties, and Social Movements. Cambridge: Cambridge University Press.

[B27] Goodwyn L. (1978). The Populist Moment. A Short History of the Agrarian Revolt in America. Oxford: Oxford University Press.

[B28] Greven T. (2016). The Rise of Right-Wing Populism in Europe and the United States. Berlin: Friedrich Ebert Foundation.

[B29] Guillén A. M. Pavolini E. (2015). Welfare states under strain in Southern Europe: overview of the special issue. Eur. J. Soc. Sec. 17, 147–157. 10.1177/138826271501700201

[B30] Hartleb F. (2015). Here to stay: anti-establishment parties in Europe. Eur. View 14, 39–49. 10.1007/s12290-015-0348-4

[B31] Hernández E. Kriesi H. (2016). The electoral consequences of the financial and economic crisis in Europe. Eur. J. Polit. Res. 55, 203–224. 10.1111/1475-6765.12122

[B32] Hobolt S. B. Tilley J. (2016). Fleeing the centre: the rise of challenger parties in the aftermath of the euro crisis. West Eur. Polit. 39, 971–991. 10.1080/01402382.2016.1181871

[B33] Huber R. A. Schimpf C. H. (2017). On the distinct effects of left-wing and right-wing populism on democratic quality. Polit. Gov. 5, 146–165. 10.17645/pag.v5i4.919

[B34] Inglehart R. F. Norris P. (2016). Trump, Brexit, and the Rise of populism: Economic have-nots and cultural backlash. HKS Faculty Research Working Paper Series. Available online at: https://research.hks.harvard.edu/publications/workingpapers/Index.aspx

[B35] Ivaldi G. Lanzone M. E. Woods D. (2017). Varieties of populism across a left-right spectrum: the case of the front national, the northern league, podemos and five star movement. Swiss Polit. Sci. Rev. 23, 354–376. 10.1111/spsr.12278

[B36] Kioupkiolis A. (2016). Podemos: the ambiguous promises of left-wing populism in contemporary Spain. J. Polit. Ideol. 21, 99–120. 10.1080/13569317.2016.1150136

[B37] Koppetsch C. (2019). Die Gesellschaft des Zorns. Rechtspopulismus im globalen Zeitalter. Bielefeld: Transcript.

[B38] Kriesi H. (2014). The populist challenge. West Eur. Polit. 37, 361–378. 10.1080/01402382.2014.887879

[B39] Kriesi H. Pappas T. S. (Eds.). (2015). European Populism in the Shadow of the Great Recession. Colchester: ECPR Press.

[B40] Laclau E. (2005). On Populist Reason. London: Verso.

[B41] Laclau E. Mouffe C. (1985). Hegemony and socialist strategy: towards a radical democratic politics. London: Verso.

[B42] Lees C. (2018). The ‘Alternative for Germany': the rise of right-wing populism at the heart of Europe. Politics 38, 295–310. 10.1177/0263395718777718

[B43] Mair P. (2006). Ruling the void? The hollowing of Western democracy. New Left Rev. 42, 25–51. Available online at: http://hdl.handle.net/1814/6418

[B44] Mair P. (2013). Ruling the Void: The Hollowing of Western Democracy. London; New York, NY: Verso Trade.

[B45] Manin B. (Ed.). (1997). “Metamorphoses of representative government,” in The Principles of Representative Government (Cambridge: Cambridge University Press), 193–235.

[B46] Manow P. (2018). Die Politische Ökonomie des Populismus. Berlin: Suhrkamp.

[B47] March L. (2017). Left and right populism compared: the British case. Br. J. Polit. Int. Relat. 19, 282–303. 10.1177/1369148117701753

[B48] Maxwell J. A. (2004). Qualitative Research Design. An Interactive Approach. Thousand Oaks, CA: SAGE Publications.

[B49] Moffitt B. (2016). The Global Rise Of Populism: Performance, Political Style, And Representation. Stanford, CA: Stanford University Press.

[B50] Moffitt B. Tormey S. (2014). Rethinking populism: politics, mediatisation and political style. Political Studies, 62, 381–397. 10.1111/1467-9248.12032

[B51] Mouffe C. (2018). For a Left Populism. London; New York, NY: Verso Books.

[B52] Mouffe C. (2019). The populist moment. Simbiót. Rev. Eletrôn. 6, 6–11.

[B53] Mudde C. (2017). “Populism: an ideational approach,” in The Oxford Handbook of Populism, eds Kaltwasser C. R. Taggart P. Ochoa Espejo P. Ostiguy P. (Oxford: Oxford University Press), 27–47.

[B54] Mudde C. Kaltwasser C. R. (Eds.). (2012). Populism in Europe and the Americas: Threat or Corrective for Democracy? Cambridge: Cambridge University Press.

[B55] Muller J.-W. (2011). Contesting Democracy: Political Ideas in Twentieth-Century Europe. New Haven, CT: Yale University Press.

[B56] Nohl A.-M. (2010). “Narrative interview and documentary interpretation,” in Qualitative analysis and documentary method in international educational research, eds Bohnsack R. Pfaff N. Weller W. (Opladen: B. Budrich, 195–217.

[B57] Olsen J. (2018). The left party and the afd: populist competitors in eastern germany. German Polit. Soc. 36, 70–83. 10.3167/gps.2018.360104

[B58] Otjes S. Louwerse T. (2015). Populists in parliament: comparing left-wing and right-wing populism in the netherlands. Polit. Stud. 63, 60–79. 10.1111/1467-9248.12089

[B59] Patton D. F. (2017). Monday, monday: eastern protest movements and German party politics since 1989. German Polit. 26, 480–497. 10.1080/09644008.2017.1365136

[B60] Piketty T. (2013). Capital in the 21st Century. Cambridge, MA: Harvard University.

[B61] Plescia C. Kritzinger S. De Sio L. (2019). Filling the void? political responsiveness of populist parties. Representation 55, 513–533. 10.1080/00344893.2019.1635197

[B62] Rama J. Santana A. (2019). In the name of the people: left populists versus right populists. Eur. Polit. Soc. 21, 17–35. 10.1080/23745118.2019.1596583

[B63] Ramiro L. (2016). Support for radical left parties in Western Europe: social background, ideology and political orientations. Eur. Polit. Sci. Rev. 8, 1–23. 10.1017/S1755773914000368

[B64] Ramiro L. Gomez R. (2017). Radical-left populism during the great recession: Podemos and its competition with the established radical left. Polit. Stud. 65, 108–126. 10.1177/0032321716647400

[B65] Rensmann L. (2018). Radical right-wing populists in parliament: examining the alternative for germany in european context. German Polit. Soc. 36, 41–73. 10.3167/gps.2018.360303

[B66] Rodrik D. (2018). Populism and the economics of globalization. J. Int. Bus. Policy 1, 12–33. 10.1057/s42214-018-0001-4

[B67] Rooduijn M. (2018). What unites the voter bases of populist parties? Comparing the electorates of 15 populist parties. Eur. Polit. Sci. Rev. 10, 351–368. 10.1017/S1755773917000145

[B68] Rucht D. (2019). Aufstehen mit oder ohne# aufstehen? Forsch. J. Soz. Beweg. 32, 8–18. 10.1515/fjsb-2019-0002

[B69] Salmela M. von Scheve C. (2017). Emotional roots of right-wing political populism. Soc. Sci. Inform. 56, 567–595. 10.1177/0539018417734419

[B70] Schmidtke O. (2002). The Third Way and the Quest for Social Justice. The Normative Claims and Policy Initiatives of the New Left in Western Europe and North America at the End of the 20th Century. Aldershot: Ashgate.

[B71] Schmidtke O. (2016). The ‘Party for Immigrants'? Social democrats' struggle with an inconvenient electoral issue. German Polit. 25, 398–413. 10.1080/09644008.2016.1182992

[B72] Stanley B. (2008). The thin ideology of populism. J. Polit. Ideol. 13, 95–110. 10.1080/13569310701822289

[B73] Stavrakakis Y. (2014). The return of “the people”: populism and anti-populism in the shadow of the european crisis. Constellations 21, 505–517. 10.1111/1467-8675.12127

[B74] Stavrakakis Y. Katsambekis G. Kioupkiolis A. Nikisianis N. Siomos T. (2018). Populism, anti-populism and crisis. Contemp. Polit. Theory 17, 4–27. 10.1057/s41296-017-0142-y

[B75] Vorländer H. Herold M. Schäller S. (2018). PEGIDA and New Right-Wing Populism in Germany. Cham: Palgrave Macmillian.

